# *Triatoma maculata*, the Vector of *Trypanosoma cruzi*, in Venezuela. Phenotypic and Genotypic Variability as Potential Indicator of Vector Displacement into the Domestic Habitat

**DOI:** 10.3389/fpubh.2014.00170

**Published:** 2014-09-30

**Authors:** Roberto García-Alzate, Daisy Lozano-Arias, Rafael Matías Reyes-Lugo, Antonio Morocoima, Leidi Herrera, Alexis Mendoza-León

**Affiliations:** ^1^Facultad de Ciencias, Instituto de Biología Experimental (IBE), Universidad Central de Venezuela, Caracas, Venezuela; ^2^Facultad de Ciencias, Instituto de Zoología & Ecología Tropical (IZET), Universidad Central de Venezuela, Caracas, Venezuela; ^3^Facultad de Medicina, Instituto de Medicina Tropical (IMT), Universidad Central de Venezuela, Caracas, Venezuela; ^4^Instituto de Medicina Tropical (IMT), Universidad de Oriente, Cumana, Venezuela

**Keywords:** *Triatoma maculata*, *Trypanosoma cruzi*, vector, Chagas disease, epidemiology, architecture of wings, molecular markers, RFLP-PCR

## Abstract

*Triatoma maculata* is a wild vector of *Trypanosoma cruzi*, the causative agent of Chagas disease; its incursion in the domestic habitat is scant. In order to establish the possible domestic habitat of *T. maculata*, we evaluated wing variability and polymorphism of genotypic markers in subpopulations of *T. maculata* that live in different habitats in Venezuela. As markers, we used the *mtCyt* b gene, previously apply to evaluate population genetic structure in triatomine species, and the β-tubulin gene region, a marker employed to study genetic variability in *Leishmania* subgenera. Adults of *T. maculata* were captured in the period 2012–2013 at domestic, peridomestic (PD), and wild areas of towns in the Venezuelan states of Anzoátegui, Bolívar, Portuguesa, Monagas, Nueva Esparta, and Sucre. The phenotypic analysis was conducted through the determination of the isometric size and conformation of the left wing of each insect (492 individuals), using the MorphoJ program. Results reveal that insects of the domestic habitat showed significant reductions in wing size and variations in anatomical characteristics associated with flying, in relation to the PD and wild habitats. The largest variability was found in Anzoátegui and Monagas. The genotypic variability was assessed by *in silico* sequence comparison of the molecular markers and PCR-RFLP assays, demonstrating a marked polymorphism for the markers in insects of the domestic habitat in comparison with the other habitats. The highest polymorphism was found for the β-tubulin marker with enzymes *Bam*HI and *Kpn*I. Additionally, the infection rate by *T. cruzi* was higher in Monagas and Sucre (26.8 and 37.0%, respectively), while in domestic habitats the infestation rate was highest in Anzoátegui (22.3%). Results suggest domestic habitat colonization by *T. maculata* that in epidemiological terms, coupled with the presence in this habitat of nymphs of the vector, represents a high risk of transmission of Chagas disease.

## Introduction

Triatomines (Hemiptera, Reduviidae, Triatominae) are blood-sucking insects that act as vectors of tripanosomatids such as *Trypanosoma rangeli* and *T. cruzi* (Kinetoplastida, Trypanosomatidae), the latter being the causal agent of American trypanosomiasis or Chagas disease. This is one of the parasitic diseases of great medical importance in the Neotropics. Chagas disease remains a public health problem in America, being distributed from the central-southern region of the United States to Southern Argentina and Chile; patients with this disease have been found in Canada and some European countries (1, 2).

Transmission of Chagas disease in Venezuela and elsewhere in South America has been traditionally associated with the domestic (D) and peridomestic (PD) environments in rural areas with poor socioeconomic conditions and high presence of vectors. However, colonization by triatomines such as *Triatoma maculata* and *Panstrongylus geniculatus* of D environments has increased, whereas before these triatomines had been mostly associated with PD or wild (S) habitats (3). In Venezuela, *T. maculata* is found in most of the states comprising the country, with the exception of Táchira and Delta Amacuro. Distribution is established from 0 to 1,500 m of altitude, with natural habitats such as palms, dry trees, fences and bird nests, and rates of infection with *T. cruzi* lower than those recorded for *Rhodnius prolixus*. Apparently, as a result of anthropogenic changes, the characteristic habitats of *T. maculata* have changed the insect becoming domestic (4).

The domiciliation of triatomines seems to be an event that can sometimes lead to the simplification of genotypic and phenotypic characteristics, which can be adaptive to macro- and microclimatic variations and reduction of wildlife mammals that serve as blood source, among others factors. These factors favor the dispersion and increase triatomine populations in anthropogenic niches (5, 6). There is suggestive evidence of a recent increase in adaptive capacity of *T. maculata* in populated areas, hence the importance of studying this vector (7).

The taxonomic position of triatomines has been revised through phenotypic studies using various methodologies, such as analysis of biochemical markers, e.g., isoenzymes, or morphometric techniques, e.g., variability analysis of the size and shape of anatomical structures (8–12), and genotypic assessment methods of polymorphism of genetic markers, e.g., the mitochondrial cytochrome *b* (mt*Cyt* b) sequence (13–15) and the ribosomal spacer region (ITS-2), among others. All of these methodologies have shown interspecific variability in different triatomines species and have been used to evaluate population genetic structure. The geometric morphometry analysis, which allowed differentiating domestic and wild insects of medical importance such as mosquitoes, and ontogenetic studies of triatomine populations have been useful in discriminating vectors, which cannot be identified by morphological or molecular variability studies (16–18).

The combined use of phenotypic methods such as geometric morphometry analysis and assessment methods such as genetic polymorphism of molecular markers would be useful in the evaluation of vector populations related to Chagas disease and the establishment of appropriate interventions for disease control. Analysis of changes in the wings, supported by the study of molecular markers such as mt*Cyt* b, has been used in Colombia in the differentiation of species of *Rhodnius* (14). In Venezuela, comparative studies, both phenotypic and genotypic, on vectors of Chagas disease are scarce; one of these studies suggests a polymorphism related to the geographical origin of the specimens in the restriction patterns of the mt*Cyt* b gene in *T. maculata* (4).

This work assesses the dispersion of this vector throughout S, PD, and D ecotopes in several Venezuelan states using both the phenotypic and genotypic approaches, in specimens of *T. maculata* captured in different regions of Venezuela. The phenotypic approach includes a geometric morphometry study to establish wing variability; the genotypic variability was evaluated through the polymorphism of the molecular markers *mtCyt* b and the β-tubulin genes region. Previously, the β-tubulin marker has been used to establish genetic variability between *Leishmania* subgenera.

## Materials and Methods

### Study area and insects

Field work was conducted in the endemic Venezuelan states Anzoátegui, Sucre, and Monagas (east of the country); Nueva Esparta (northeast, Margarita island), Bolívar (south), and Portuguesa (west). Sampling was carried out following the method proposed by Schofield (19). Specimens of *T. maculata* were collected through a direct search by personnel previously trained; sampling was conducted twice/year in each region and the capture effort occurred at 5 h/man by day or night for 5-day visit to each region. A total of 26 locations distributed in these states were visited to collect, in periods of high and low precipitation, specimens of *T. maculata*, directly from ecotopes defined following previous criteria. In D habitat, attention was put on internal walls of the houses, rooms, roofs, furniture, and in the ceiling; external domiciles or PD habitat (30 m around the exterior walls of the house), the area explored included farmyards, henhouses, and wood piles; and in S habitat (removed 30 m from the PD habitat), the area explored covered palm trees, tree holes, cave, and crops (20, 21). A total of 492 adult insects, male (M) and female (F), were collected and used in this study. Three indices were calculated; the colonization index was determined as CI (%) = 100 × total numbers of houses presenting nymphs/total number of houses with adults; the dispersion as DI (%) = 100 × number of locations with adults/number of locations studied, and infection index as II (%) = 100 × number of adults infected with *T. cruzi*/total number of adults captured. Specimens were dissected and their intestinal contents and/or hemolymph examined under the microscope for the presence of *Trypanosoma*; samples of the intestinal contents were used to evaluate the presence of *T. cruzi* and *T. rangeli* by means of a PCR assay (22). After dissection, the collected specimens were preserved in 70% alcohol and stored at −20°C for further analysis. Colonies from each location were established in the laboratory for morphometry analysis.

### Geometric morphometry analysis: metric data, shape, and size variation

**Figure 1:**
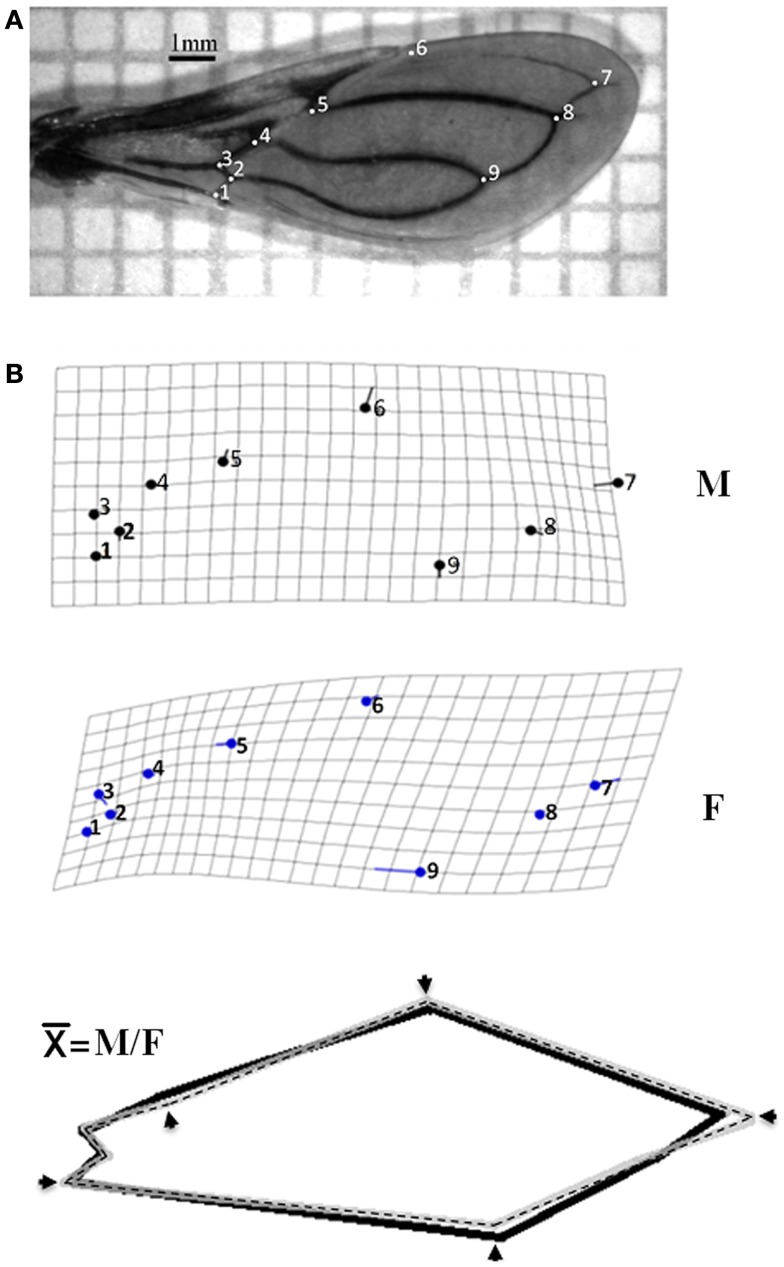
**Differences in wing shape between genders of *Triatoma maculata* from Venezuela**. **(A)** Landmark points type I identified in wing of *T. maculata*. Numbering of points (PAR 1–9) refer to the arrangement followed to obtain the coordinates using *tps* Dig 2.0. **(B)** Differences in wing shape architecture of *T. maculata*. Differences in wing shape between male (M) and female (F) of *T. maculata* are represented by grids deformation and variation between homologous landmark (solid circles). After superposition to the homologous consensus ($\bar{\text{X}}$) between M (solid lane) and F (dashed lane), the differences in wing shape are represented by incongruence between homologous landmarks (arrows).

For each individual, only the left wing was examined and included in the analysis. The wings were mounted between microscopy slides and cover-slips and photographed using a digital camera Leica S6D. Nine landmarks (Figure 1A) were identified in each wing according to previous references (18, 23); the geometric coordinates of each landmark were digitized and shape variables (partial warps) were obtained using *tpsDig* Version 2 (24). Data were submitted to a discriminant analysis to examine differences in wing shape between male (M) and female (F) and statistical significant was evaluate by the Wilk’s lambda statistics. For comparison of wing size between genders and among ecotopes within each gender, we used the isometric estimator centroid size (CS) derived from coordinate data (10, 15, 23). Statistical analysis was carried out using the MorphoJ software package for geometric morphometric (25). Landmark coordinate (*x, y*) configurations were registered and aligned using the Procrustes analysis and covariance analysis was implemented with proportions of re-classified groups and MANOVA. Then, wing shape variable and the CS were analyzed using the principal component analysis. For this analysis, M and F were processed separately due to the sexual size dimorphism of Triatominae. The relationship between shape and size was explored by a regression analysis. The significance of wing conformation due to landmark variation was established using the canonical variate analysis.

### DNA extraction and molecular marker amplification

Total genomic DNA was extracted from all six legs of each specimen using the Wizard Genomic kit (Promega, Madison, WI, USA. Cat. No. A1620). Purity and integrity of the DNA were determined by agarose gel electrophoresis. The same procedure was used to isolate the DNA from triatomine intestinal content. The extracted genomic DNA was resuspended in TE buffer (10 mM Tris, pH 7.4 and 1 mM EDTA), and stored at 4°C for further analysis.

For direct molecular identification of *T. cruzi* and *T. rangeli*, DNA isolated from the intestinal content of insects was used to amplify the variable region of the minicircles of kinetoplast DNA (kDNA) and the non-transcribed spacer region of the mini-exon (26, 27).

Two markers, previously reported as fit to evaluate genetic diversity in different organisms, were used in *T. maculata*. First, the mt*Cyt* b gene apply to evaluate population genetic structure in different triatomine species (28, 29), and second, the β-tubulin gene region, a marker employ to study genetic variability in *Leishmania* subgenera (30, 31).

The β-tubulin primers were designed by multiplex alignment of similar genes available in the NCBI Genbank from *Triatoma tibiamaculata* (KC249297), *T. infestans* (JK33877), *T. braziliensis* (EC917343), *Rhodnius prolixus* (FG544591), *Aedes aegypti* (XM00165064), and *Drosophila melanogaster* (AE0135994), and after bioinformatics sequence analysis, the primers sequences TubTmf and TubTmr were selected (Table 1).

**Table 1:** **Molecular markers, primers, and PCR assay conditions**.

Marker	Primers 5′–3′	PCR assay cycles	Fragment size (pb)	Reference
mt*Cyt* b	***CYT BF* 135** GGACAAATATCATGAGGAGCAACAG	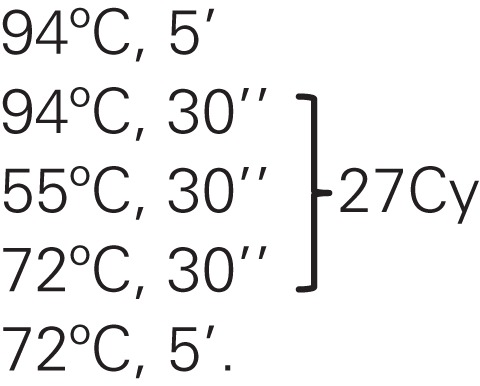	600	(28, 29)
	***CYT BR* 135** ATTACTCCTCCTAGCTTATTAGGAATTG	
β-tubulin	***TUB TMr*** GACACGCAGCGCTTGCGCACTCGT	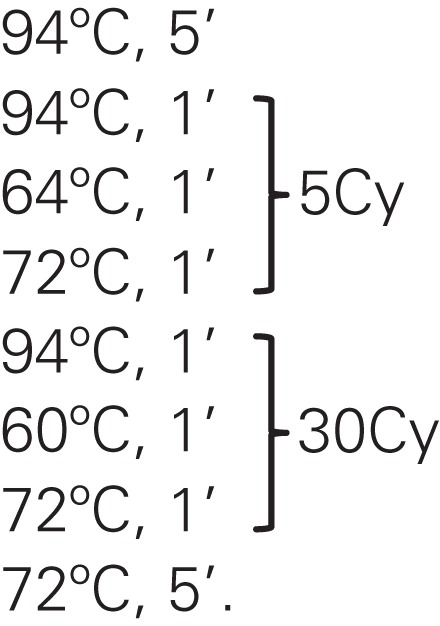	980	(31)
	***TUB TMf*** CCCGTCCTGCCTCGCCTGC	

*Cy = number of cycles; ′ = min; ″ = s*.

Markers were amplified under standard polymerase chain reaction (PCR) assays as described previously; the reaction was carried out in a final volume of 25 μl containing 12.5 μl cocktail of PCR mix 2X (GoTaq Master Mix, Promega, Madison, WI, USA. Cat.# M7122), 0.4 μmol primers (stock 100 μM) and 5 ng total genomic DNA; the PCR reaction was performed in an MJ Research PTC-200 thermocycler.

### PCR-RFLP of the β-tubulin gene marker

The PCR fragment of the β-tubulin gene marker from specimens of different ecotopes was partially sequenced using the *Sequence Navigator* version 1.0.1 (Perkin Elmer Applied Biosystem) and the *in silico* restriction map was established (NETcutter version II). The endonucleases *Bam*HI and *Kpn*I (Life Technology) were selected for double digestion of the β-tubulin-PCR product following the manufacturer’s instructions, and the digested DNA fragments fractionated by agarose gel electrophoresis.

### Electrophoresis

The purity and integrity of the DNA were determined by electrophoresis in 0.6% agarose gel at 80 V for 1 h in TBE buffer (90 mM Tris-HCl, pH 8.0; 90 mM boric acid; 2.5 mM EDTA). The PCR products were analyzed by electrophoresis on 1.5% agarose gel in TBE buffer and the RFLP products were subjected to electrophoresis in 3% agarose gel in TBE buffer. After electrophoresis, the gel was stained with ethidium bromide, visualized with UV illumination, and recorded on a gel documentation system.

### Genetic diversity

The relationships, genetic differentiation, among the pattern fragments of the RFLP analysis of the β-tubulin marker (presence or absence of fragments) from different states and ecotopes were estimated using the NJ algorithm and the tree is based on a Kimura 2-parameter distance matrix (32, 33). Statistical support for branches in the NJ tree was assessed by the bootstrap method with 1,000 replicates. The analysis was conducted using the software MEGA V.4 (34).

## Results

### Phenotypic variability

#### Colonization and infection of *T. maculata* with *T. cruzi*

Of the 492 specimens of *T. macula*, 49.2% were M; the insects were collected from six Venezuelan states, whose distribution by location showed a majority of these specimens distributed in the PD (67.07%) and domestic (26%) ecotopes, followed to a lesser extent by the wild ecotopes (7.3%). On average, 20.73% of these specimens were positive for *T. cruzi* according to the specific kDNA and mini-exon-PCR assays (Table 2). The higher CI found in Anzoátegui and Monagas states in relation to the other states coupled to a high II to *T. cruzi* in these two states suggests a higher rate of household colonization and showed the importance of *T. maculata* as a vector.

**Table 2:** **Geographical origin and ecotope of *Triatoma maculata***.

State^a^ (total/F/M)	Location	Coordinates		Ecotopes			CI (%)	II (%)
		N	W	D	PD	S	
**Anzoátegui (284/134/150)**	Pico de Neverí	09°75′	065°02′	0	13	0	22.3	18.4
	El Enial	09°79′	065°02′	0	33	4	
	San José de las Margaritas del Llano	09°79′	065°02′	33	94	5	
	Los Ranchos	10°23′	064°60′	29	40	0	
	Guastrantal	10°11′	064°59′	0	23	7	
	Mundo nuevo	09°30′	064°35′	1	2	0	
**Monagas (86/52/34)**	Caripito	09°98′	063°49′	9	4	8	13.81	26.8
	Aragua de Maturín	09°77′	063°15′	6	29	3	
	Musu	09°61′	063°08′	8	5	5	
	La Planchada	09°93′	063°40′	0	7	0	
**Portuguesa (63/37/26)**	Jabillal	09°42′	069°18′	10	18	3	5.47	19.1
	Las panelas	08°58′	069°58′	6	20	6	
**Sucre (29/15/14)**	La Sabana	10°18′	064°21′	0	3	1	1.76	37.9
	Guayabal	10°13′	064°42′	2	4	3	
	La Piscina	10°42′	064°19′	2	6	1	
	UDO Cumana	10°46′	064°14′	0	4	3	
**Bolívar (16/7/9)**	Caruachi	08°35′	062°54′	0	2	0	8.2	18.7
	Guasipati	08°30′	062°64′	3	3	0	
	Tocoma	08°18′	062°84′	0	4	0	
	Gran sabana	08°13′	062°74′	0	3	0	
	La laguna	08°00′	062°64′	0	1	0	
**Nueva Esparta (8/3/5)**	Porlamar	10°95′	063°88′	0	2	0	0	12.5
	Roble	11°06′	063°84′	0	2	0	
	Fuentidueño	10°90′	063°96′	0	1	0	
	La sierra	10°99′	063°91′	0	3	0	

*^a^Venezuela states*.

*N, total number of insects; F, female; M, male; D, domestic; PD, peridomestic; S, wild; CI, colonization Index; II, infection index*.

#### Size and shape variation

Average size of the membrane region of the wing was 1,984 mm for F and 1,786 mm for M. The discriminate function for gender re-classified wings, 75% for M (M 30/40) and 82% for F (F 33/40); this function in turn contained 94% of gender variance, showing significant differences in the formation of the wing according to gender (Wilk’s lambda: 0.543 and 0.754 for M and F respectively; *p* < 0.001). When analyzing the intraspecific allometric effect (degree of deformation of the wing), using size (component 1) as the independent variable and wing conformation (component 2) as the dependent variable, the contribution of these components was 49.5% of the variation. The differences in conformation based on the deformation of grids made by discriminating analysis disclosed that the M has a lower degree of variation compared with F, and a clear sexual dimorphism (Figure 1B). The mean wing deformation ($\bar{\text{X}}$), obtained through the overlapping of gender-related wing deformation grids, showed changes in the landmark points; the decrease in wing size was observed in at least one of the landmarks (PAR 1–9) and in some cases by the loss of anatomical landmarks (Figure 1B). The same changes of the landmark points were found after the third filial generation in colonies from the same location established in the laboratory.

**Figure 2:**
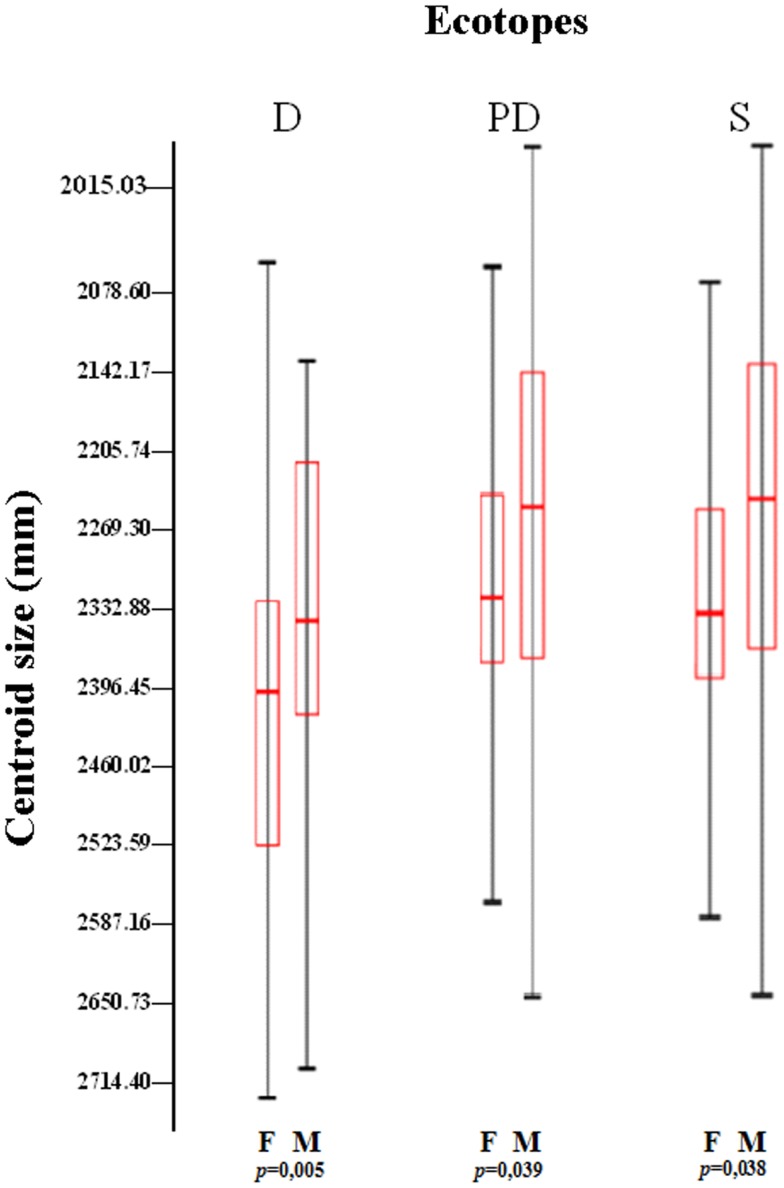
**Variation of the centroid size among ecotopes and gender of *Triatoma maculata* from Venezuela**. The boxes show the isometric size differences of wings between ecotopes (D, domestic; PD, peridomestic; and S, wild) and sex (M, male and F, female), from different states and locations. Each box shows the mean (horizontal line inside the box), standard deviation (vertical line). The number of individuals was M, 242 (D, 29; PD, 172; and S, 43) and F, 250 (D, 24; PD, 158; and S, 63). *p*: statistical significant differences.

The formation of wing architecture based on the variation of CS in each ecotope (Figure 2) evidenced an association between PD and S insects with no significant difference between them (*p* = 0.0392 for PD and *p* = 0.0382 for S), whereas significant differences (*p* = 0.0051) were observed when comparison was carried out between PD and S specimens together with insects collected in D, regardless of gender (Figure 2).

**Figure 3:**
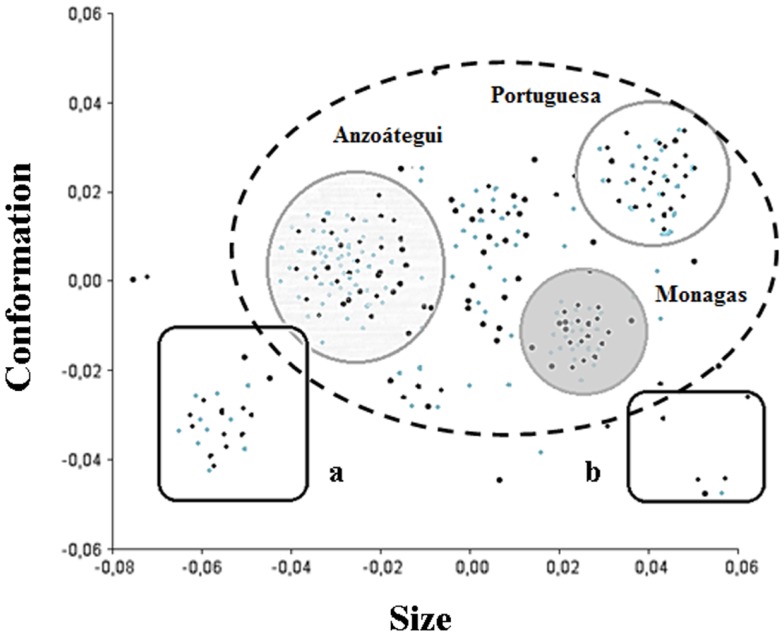
**Phenotypic variability of *Triatoma maculata***. Diagram of factorial data of the main components of wing architecture of *T. maculata*. The components size and conformation make the higher contribution (70%) in wing variability. The dashed line circle represents the standard group, whereas the solid line ones represent groupings by confidence ellipses (95%) of male (black points) and female (gray points) specimens from Anzoátegui, Monagas, and Portuguese states. The boxes represent the grouping of M and F specimens that showed the greatest differences in wing size and shape when compared to the consensus configuration.

The variation in wing size and conformation allowed the grouping of three states (Figure 3). The results showed clusters of M and F of *T. maculata* captured in S and PD ecotopes in Anzoátegui, Monagas, and Portuguesa. Interestingly, higher variation in the consensus tendency was found in both genders in specimens captured in D ecotopes of Anzoátegui and Monagas (Figure 3, squares a and b).

### Genetic diversity

#### Mini-exon analysis

Twenty percent of specimens were identified as positive for *T. cruzi* infection and 1.2% presented a co-infection with *T. rangeli* as demonstrated by a PCR assay for the mini-exon. The lineage of *T. cruzi* circulating in all states was identified as TcI after amplification of a band of 200 bp from the non-transcribed region of the mini-exon (results not shown).

#### Variability of molecular markers

**Figure 4:**
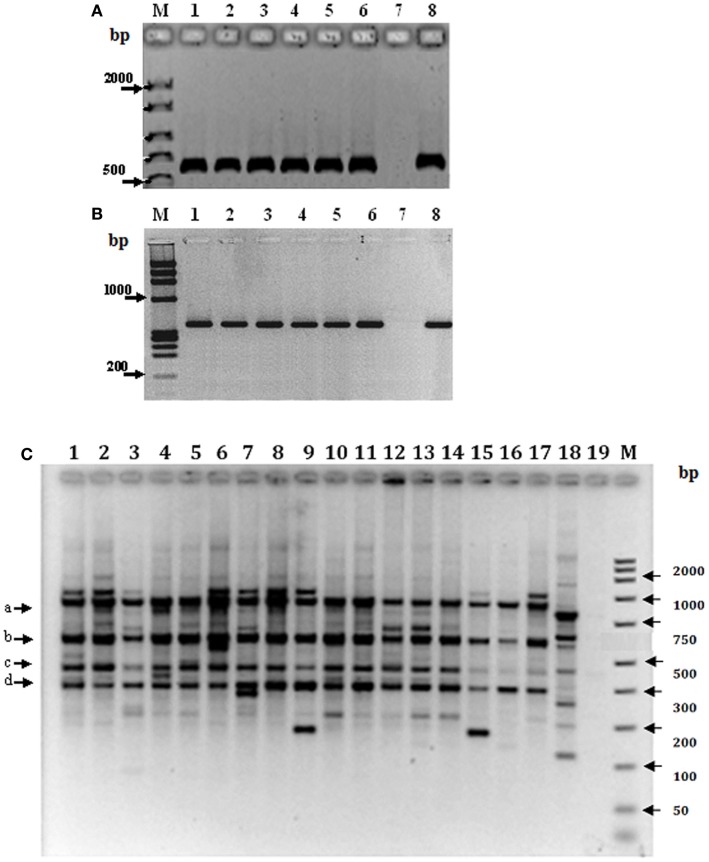
**Genetic variability of *Triatoma maculata* from Venezuela**. Amplification products of *mtCyt* b **(A)** and the β-tubulin gene region (**B**). Lanes 1–6 in **(A,B)**, Venezuelan states: Anzoátegui, Monagas, Sucre, Bolívar, Portuguesa, Nueva Esparta; lane 7, negative control without DNA and lane 8, DNA from a laboratory specimen. **(C)** RFLP analysis of the PCR product of the β-tubulin marker representative of different states, locations and ecotopes. Lanes 1–3: Anzoátegui, Los Ranchos, lane 1 D; lane 2, PD; and Guastrantal, lane 3 S; lanes 4–6: Sucre, Guayabal, lane 4 D; lane 5 PD; and La Piscina, lane 6 S; lanes 7–9: Monagas, Caripito, lane 7 D; lane 8 PD; and lane 9 S; lanes 10–12: Bolívar, Guasipati, lane 10 D; lanes 11 and 12 PD; lane 13–15: Nueva Esparta, La Sierra, lanes 13 and 14 PD; and lane 15 S; lanes 16–18: Portuguesa, Las Panelas, lane 16 D; lane 17 PD; and lane 18 S. Lane 19: negative control (no DNA). M: 100 bp (GIBCO BRL) as molecular marker.

Genomic DNA from specimens of *T. maculata* representative of each state, regardless of location or ecotope, was evaluated by amplification of mt*Cyt* b and the β-tubulin gene region (Table 1; Figure 4). The results showed a unique PCR product of 600 bp for mt*Cyt* b (Figure 4A), which was common in size among specimens of different locations of different states and also among the ecotopes of these locations; the low variability of this product, as determined by partial sequencing, did not allow to establish differences based on mt*Cyt* b between specimens (results non-shown). In contrast, a unique fragment of 980 bp was generated by the amplification of the β-tubulin marker (Figure 4B); the partial sequencing of this fragment showed differences between states and ecotopes, suggesting genetic variability in populations of *T. maculata*.

In order to establish the variability of the β-tubulin gene marker, the PCR product from specimens of different states and ecotopes was sequenced and *in silico* restriction fragment maps were established and used to identify the restriction enzymes to be used to evaluate genetic differences among specimens from different ecotopes using RFLP analysis. The results revealed a partial common pattern for double digestion with *Bam*HI-*Kpn*I, with quantitative and qualitative differences among specimens of the majority of states, represented for bands of 980 (**a**), 620 (**b**), 450 (**c**), and 300 bp (**d**) (Figure 4C). This pattern was independent of location or ecotope, the exception being Portuguesa state, where differences were found between ecotopes, since in this location only band (**c**) was present in the S pattern. In addition, other individual fragments were observed among the different ecotopes in all states; however, differences between ecotopes of the same state were evident, e.g., Anzoátegui, Sucre, Monagas, and Portuguesa (Figure 4C, lanes 1–9 and 16–18), as well as between the same ecotope when different states were compared (Figure 4C, lanes 1, 4, 7, and 16).

**Figure 5:**
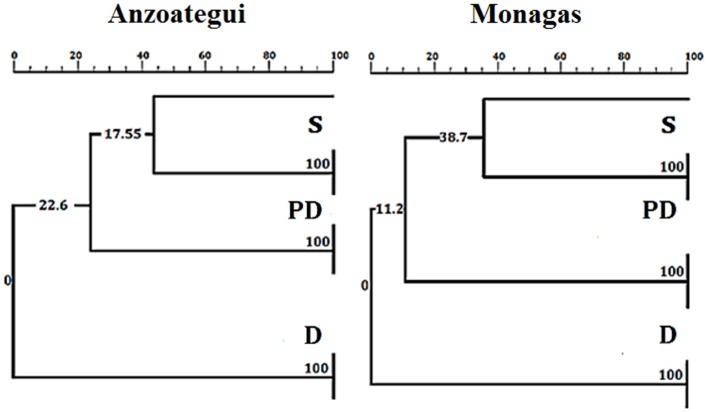
**Neighbor-joining tree based on Kimura 2-parameters of RFLP fragments of the β-tubulin marker of *Triatoma maculata***. The numbers on the branches indicate boostrap support. D, PD, and S: domestic, peridomestic, and wild ecotopes. The same tree as for Monagas was found for Sucre, Bolívar, Nueva Esparta, and Portuguesa states.

The PCR-RFLP patterns obtained for the region of β-tubulin (Figure 4C) were evaluated according to the presence or absence of bands to establish comparative marker variability among different states and particularly among ecotopes. Anzoátegui and Monagas states had the largest differences in pattern by ecotopes (genetic differentiation index, Fst 0.476, *p* = 0.019). Anzoátegui state showed a 60% similarity between PD and S ecotopes, and the remaining 40% was due to D ecotope. The rest of the states revealed similar groupings in their genetic profiles (Figure 4C). This suggests that the restriction patterns obtained could be an alternative for intraspecific differentiation of *T. maculata* associated with different ecotopes. Comparative analysis of data from Anzoátegui and Monagas states showed that about 60% of the restriction fragments are common regardless of ecotope. Maximum Parsimony analysis with 1,000 replicates, statistically supported by bootstrapping, generating a similar clustering among specimens of S and PD ecotopes, which would indicate similarity, while the genetic pattern of D differs, causing it to clump as a synapomorphic group (Figure 5), separated according to ecotopes.

## Discussion

The present study demonstrated for the first time domiciliary adaptation processes of *T. maculata* in several Venezuelan states, using two approaches, phenotypic as the geometric morphometry of wing, and genotypic as the variability of the β-tubulin molecular marker. This, together with the presence of nymphs in houses and a high rate of infection with *T. cruzi* of specimens captured inside the home indicates that there is increased risk factor in the transmission of Chagas disease in Venezuela. Our results showed that there is discrimination of *T. maculata* according to its ecotopes, since specimens from S and PD ecotopes were more similar between them in wing architecture and variability of the β-tubulin marker in comparison with that from D ecotope. Previous studies suggest that *T. maculata* is contributing to increased risk of transmission of *T. cruzi* in the human population from various regions of Venezuela, particularly in the north-eastern region where specimens of this species showed a high percentage of infestation and a high rate of infection with *T. cruzi* (35).

Our results demonstrate the utility of geometric morphometry study of wing architecture to establish sexual dimorphism, phenotypic variability, and the association of these variables to different ecotopes of *T. maculata*. Thus, this is a robust tool to determine intraspecific differences possibly related to the geographical distribution in the macro- and microenvironment.

It has been demonstrated that Chagas disease vectors traditionally considered exclusively S have the possibility to change their behavior and colonize D habitat, with a high risk in the epidemiology of the disease (36). The association with particular habitats or ecotopes of different phenotypical and genotypical characters in triatomines has proved important in vector identification, dispersion, and colonization properties and in general in the epidemiology and control of Chagas disease in South America (14, 37–39).

Different molecular strategies have been used to study genetic diversity in populations of triatomine vectors, such as variability of the mt*Cyt* b gene, microsatellites, and random amplified polymorphic DNA (RAPD) (14, 28, 37, 40). Previous studies on the genetic variability of *T. maculata* in Venezuela using mt*Cyt* b partially enabled to infer the occurrence of different haplotypes for populations in Anzoátegui and Portuguesa (4). In our study, mt*Cyt* b presented the lowest level of variability, and these variations failed to discriminate *T. maculata* by ecotopes.

The polymorphism for β-tubulin marker in specimens of *T. maculata* generated evident variations between ecotopes of different locations of the Venezuelan states. This was particularly seven haplotypes for samples collected in homes and five haplotypes for copies of PD and wild ecotopes. The correspondence observed between phenotypic and genotypic grouping indicates that the joint application of both approaches is a robust tool for the study of vector domiciliation.

This is the first time that the molecular marker of β-tubulin was used for evaluation of the genetic variability in *T. maculata*, the evaluation of this sequence being important in establishing it for use as a valuable tool in the genetic evaluation of triatomine population.

## Conclusion

This work presents for the first time the relationship between phenotypic and genotypic approaches in the discrimination of *T. maculata*, a vector of Chagas disease, according to its ecotopes in Venezuela. Wing architecture and variability of the β-tubulin DNA region, the variables used to differentiate *T. maculata* populations, showed that specimens from S and PD ecotopes were more similar between them than those captured in the D ecotope which, together with the presence of nymphs in the houses and a high rate of infection with *T. cruzi* of D specimens, increases the risk factor in the transmission of Chagas disease in Venezuela. Eradication of the vector in the domestic ecotopes followed by vigilance of re-infection will be important in reducing transmission of Chagas disease; however, integrative research is necessary to understand the vector population structure, domiciliation, and parasitic transmission.

## Author Contributions

Leidi Herrera (Coordinator), Roberto García-Alzate, Antonio Morocoima, and Rafael Matías Reyes-Lugo, designed the field work. Roberto García-Alzate, Daisy Lozano-Arias, Leidi Herrera, and Alexis Mendoza-León performed analyses. Roberto García-Alzate, Daisy Lozano-Arias, and Alexis Mendoza-León analyzed the data and prepared the manuscript. All authors read and approved the final manuscript.

## Conflict of Interest Statement

The authors declare that the research was conducted in the absence of any commercial or financial relationships that could be construed as a potential conflict of interest.
